# Findings of Case-Study Analysis: System-Level Biomimicry in Built-Environment Design

**DOI:** 10.3390/biomimetics4040073

**Published:** 2019-11-01

**Authors:** Samantha Hayes, Cheryl Desha, Mark Gibbs

**Affiliations:** 1 Griffith University Cities Research Institute, Brisbane 4111, Australia; 2 Queensland University of Technology, Institute for Future Environments, Brisbane 4000, Australia

**Keywords:** biomimicry, built environment, infrastructure, systems, ecological performance standards

## Abstract

Complex systems challenges like those facing 21st-century humanity, require system-level solutions that avoid siloed or unnecessarily narrow responses. System-level biomimicry aims to identify and adopt design approaches that have been developed and refined within ecosystems over 3.8 billion years of evolution. While not new, system-level biomimetic solutions have been less widely applied in urban design than the ‘form’ and ‘process’ level counterparts. This paper explores insights from a selection of system-level case studies in the built environment, using meta-analysis to investigate common challenges and priorities from these projects to support knowledge-sharing and continued development in the field. Using a grounded research approach, common themes are distilled, and findings presented regarding success and barriers to implementation and scaling. Considering the findings, and drawing on complex adaptive systems theory, the paper posits opportunities to facilitate broader implementation and mainstreaming of system-level biomimetic design approaches in the built environment.

## 1. Introduction

It is well recognised that complex systems challenges require system-level solutions that avoid siloed or unnecessarily narrow responses, and this is particularly relevant for urban design and built-environment applications, where there has been a tendency towards considering individual buildings, infrastructure assets or projects as discrete and isolated entities. Drawing upon decades of research into biological design approaches, principles and relationships between organisms within ecosystems, biomimicry aims to identify and abstract design approaches that have developed within ecosystems over 3.8 billion years of evolution. The ‘systems’ approach to biomimicry, while not new, has arguably been less widely adopted within built-environment applications than its ‘form’ and ‘process’ level counterparts, where organisms have been investigated and elements of their form or processes (self-repair, self-assembly, multi-functionality) translated to a wealth of materials, products and design approaches. In a recent systematic literature review of biomimicry research for built-environment applications, Hayes et al. [1] highlighted the relatively limited focus on system-level approaches to biomimetic design in the built environment, noting the potential implications of this on objectives of regenerative design, sustainability and resilience. Such limitations are not restricted to building and infrastructure applications, and have been explored in work by Reap [2] and Pedersen Zari and Storey [3] among others.

Systems-level biomimicry, when considered from a complex systems perspective, still draws on optimized design strategies at the organism level, while recognizing that (a) organisms’ characteristics and design strategies have emerged in the context of complex adaptive systems and will continue to adapt over time, and (b) that the contexts into which biomimicry approaches are applied are also complex socio-eco-technological systems. While published research into systems-level biomimicry is less common, there are several projects underway both in the public and private sector, working to further define, refine and test biomimetic design approaches in the built environment. This paper synthesises findings from a meta-analysis of case-study projects that have used system-level biomimetic design tools and frameworks in built-environment projects. It investigates common challenges, benefits and key learning from these projects to support knowledge-sharing and continued development in the field.

### 1.1. Sustainability, Resilience and Biomimicry in the Built Environment

Built-environment design is changing, with global shifts (albeit incremental) towards sustainable development including the emergence of sustainability rating schemes, adjustments to building codes and specifications, and shifting government and corporate policy [4,5]. As these approaches increasingly become standardized and integrated into business-as-usual practice, leading organisations and champions are looking towards ‘next steps’ in sustainable design and construction, identifying emerging opportunities for innovation and leadership [6,7,8]. At this leading edge, concepts of sustainability and resilience continue to be challenged and advanced, with efforts to shift practice from incremental and often narrow interpretations to more holistic systems-level perspectives that recognise complexity and uncertainty [9,10]. For sustainability efforts, this means moving beyond ‘damage reduction’ and instead towards regenerative design [11,12,13]. This includes recognition that urban design occurs within complex adaptive systems, where concepts of emergence and evolution apply not only to ecosystems but also to the built environment [14].

For resilience, it includes a shift from disaster risk reduction and ‘fail-safe’ approaches to robustness, and towards understandings of resilience that align with socio-ecological systems, such as ‘antifragility’ and safe-to-fail solutions with sustained adaptability [10,15,16,17]. While these shifts remain more visible in academia than industry, an increasing number of private and public sector parties are working to integrate such concepts into applied projects, recognising the importance of system-level approaches to complex systems challenges. The evolution of applied biomimicry mirrors these trends, with the role for system-level approaches increasingly recognised, yet less frequently applied in practice [1,2,18]. Recognising the potential for systems approaches to ‘innovation inspired by nature’, several frameworks are now emerging to support biomimetic design at this level.

### 1.2. System-Level Biomimicry Tools and Frameworks

At the system level, as outlined in Hayes et al. [1], three biomimetic design approaches in particular are being tested in built-environment projects – Life’s Principles (or biological design principles), Genius of Place, and Ecological Performance Standards [18,19,20]. While applied built-environment examples are limited, each project provides insight into the opportunities and challenges of translating these approaches from concept to implementation. 

‘Life’s Principles’ (LP) reflect design patterns and strategies commonly adopted by organisms within ecosystems. Distilled from a wide-ranging and iterative review of ecological literature by Baumeister et al. [19], the set of six overarching principles and the corresponding sub-elements offer a list of key characteristics for consideration in design. In some cases, the principles are used as a prompt for option identification, guiding users to consider the variety of strategies adopted within ecosystems. The principles can also be adopted as an evaluation framework, allowing designers to assess their design against each of the principles. Although some of the principles reflect more traditional sustainability concepts (e.g., ‘be resource efficient’), others push the boundaries further (e.g., ‘Use life-friendly chemistry’). There is another important distinction: these principles have been consistently identified in most organisms and ecosystems worldwide, where not one, but all of the principles are reflected repeatedly across scales, contexts and geographical locations. As such, the view is that these principles offer a guiding framework of what ‘good’ and ‘regenerative’ design looks like, and that the expectation is that designers would design for all, not just one or two, of these design principles. Rather than taking a siloed approach through consideration only of resource efficiency, for example, the systems approach looks at how the overarching design ‘evolves to survive’, ‘adapts to changing conditions’ is ‘locally attuned and responsive’ and ‘integrates development with growth’. Drawing these patterns directly from nature, designers may use them as a structure and check-in, to reflect on the extent to which their built-environment design aligns with these natural environment principles. This approach shifts away from drawing on one specific and ‘single-point-in-time’ biomimetic learning to inform a specific component of a building or asset, an approach that has typified many examples of biomimicry in design to date. Instead it moves towards a system-level understanding that learns from patterns in organisms and ecosystems to inform the overarching design approach, and where the design itself accounts for dynamic and unpredictable system pressures and changes over time. While peer-reviewed publications referencing the principles are limited, the LP tool is currently being utilised on several built-environment projects internationally. 

The ‘Genius of Place’ (GoP) framework offers a biomimetic place-based and targeted design approach, where a project team investigates the local ecosystem in order to identify key strategies and mechanisms that organisms have developed in that place in response to the specific operating conditions and challenges of that place. For example, in the extreme heat and dry conditions of the Sonoran Desert, USA, flora and fauna have developed locally attuned designs that help to manage temperature and maximize water retention. GoP investigations allow for such design strategies to be explored, analysed and translated to built-environment design strategies that mimic the functional benefits of that strategy. Recognising built assets as components of complex socio-eco-technological systems, the GoP approach supports place-based and locally attuned design that explicitly identifies opportunities for design innovation inspired by local organisms. 

Finally, the ‘Ecological Performance Standards’ (EPS) frameworks focus primarily on measuring functional performance. Building on the regenerative design agenda, EPS proposes a shift from using business-as-usual design as a baseline, to instead using ecosystem performance as a design baseline—asking the questions “What would nature do here?” and “How would an ecosystem function here?”. Here, the process is to quantify the ecosystem services that would have been generated by an intact ecosystem in that location. These metrics then become the desired performance standard for the built-environment asset to be constructed there—shifting the objective from damage reduction to regenerative performance, where the design objective is to deliver a built-environment design that is functionally indistinguishable from the ecosystem that would otherwise exist in that place. Using both existing and novel design approaches, products and technologies, the project team seek to deliver ecosystem services through planning, design, construction and operation of the asset. As documented in existing case studies, the Lavasa project in India offers one example of EPS applied to a community development, with a view to designing a community masterplan that learnt from nature to inform regenerative design at the system or community level [20]. Shifting from narrow and siloed performance metrics to ecosystem cycles and functional performance provides a foundation for more holistic and systems-based evaluation of built-environment performance.

### 1.3. Innovation in Complex Adaptive Systems

When seeking to introduce innovative approaches to challenges in complex systems, it is important to understand the systems at play both from an ecological and socio-technological perspective [21]. In transition management and innovation diffusion, it is increasingly recognized that the introduction and diffusion of innovative approaches is rarely linear, with complex system interactions, unintended side effects and feedback loops playing important roles in the eventual success of innovation diffusion [21]. Appreciating this, Geels [22] Multi Level Perspective (MLP) theory refers to three levels: technological niches, socio-technical regimes and exogenous socio-technical landscape, that should align for innovation breakthroughs to occur. Nan et al. [23] expand on the specific attributes and characteristics that support innovation diffusion in complex adaptive systems, providing a framework for analysis of the case-study projects investigated in this paper.

At the level of the technological niche or innovation, they highlight three key attributes that can influence adoption: (1) relative advantage, or the additional potential value available via the innovation; (2) network externalities, the external influences and context in which the innovation exists, including the extent to which innovation value increases as more members of the population adopt it; and (3) arduousness, the effort required by adopters in order to implement and gain value from the innovation [23]. Regarding the socio-technological regime, they offer the AMC framework on diffusion of innovations, where A is ‘Awareness’, M ‘Motivation’, and C ‘Capability’—three key factors for success in innovation diffusion. Finally, they refer to Environment, the contextual system or exogenous socio-technical landscape within which the innovation is applied. Importantly, this model includes a focus on the role of adopters (and their level of innovativeness), influencers (through dominance, specialization and exemplariness), and the interactions between these agents and within social network structures [23]. This recognition of various scale levels is integral to complex adaptive systems theory, where it is understood that emergent properties result from complex interrelationships and feedback loops across levels and scales within a system, as opposed to linear and predictable implementation pathways.

Innovation in complex systems requires flexibility, adaptability, willingness to adjust and update objectives and methods, and a reliance on, rather than avoidance of, periods of disequilibria—to avoid stagnation and allow for innovative approaches to emerge [21]. This includes creating space (including provision of resources, knowledge and access) for innovation niches, and allowing agents the flexibility to identify and explore unique approaches. While recognising inherent uncertainty in projecting future developments within complex systems, it also requires some anticipation of future trends, which is nonetheless possible through insights into broad transitions and path dependency [21]. This paper investigates applied examples of system-level biomimicry, including successes and challenges, before drawing on this understanding of complex systems to frame opportunities for further uptake and mainstreaming of biomimetic design approaches.

## 2. Materials and Methods

The investigation method was influenced by the relatively small number of publications relating to system-level biomimicry projects in the built environment. Recognising the emerging nature of system-level biomimicry frameworks, and the fact that implementation has in several cases been driven and documented by industry and government rather than peer-reviewed academic research, a grounded research method of investigation was adopted. This comprised case-study analysis, including the development of criteria, interviews and document analysis. Web-based searches revealed several relevant projects for review, and word-of-mouth referral was utilised in conversation with these project teams to identify other examples of applied system-level biomimicry tools and frameworks in the built environment.

In the course of a Research Fellowship at The Biomimicry Center at Arizona State University, the lead author engaged with biomimicry experts internationally to identify additional examples of built-environment projects incorporating system-level biomimicry into planning, design, construction and/or operation. With the emergence of additional published literature, it is anticipated that future iterations of this work may investigate relevant peer-reviewed publications to supplement the catalogue of applied projects presented in this paper.

### 2.1. Case-Study Criteria

Using the following criteria, six case-study projects were selected, with three in America, one based in America with a global scope, one as a joint study across America and Australia, and one in Africa (Table 1):

- The project is an example of biomimicry at the systems level; and
- The project includes an application of the biomimetic approach to one or more assets/locations; and
- There exists potential for replication/adaptation; and
- There is access to archival data (online/print/personnel).

Projects were also reviewed to identify evidence of biomimetic intent at project commencement, avoiding projects with incidental or ‘accidental’ biomimetic outcomes and focusing on those seeking to intentionally incorporate biomimicry into design. The following exclusion criteria were also applied:

- Computer science and information technology (IT) approaches, such as biomimetic approaches to optimising computer software and hardware systems;
- Solutions that have not yet been applied. The intent of this review was to identify current practice in implementation, particularly given the limited peer-reviewed literature available on these frameworks. As such, only projects that had been implemented (including at the planning and design phases) were included;
- Form and process level approaches, which were synthesised within a systematic literature review conducted by Hayes, Desha and Baumeister [1];
- Documents published in languages other than English; and
- Projects pursuing biophilic or bio-utilisation outcomes in lieu of biomimetic approaches.

### 2.2. Interview Method

Interviews were conducted with case-study personnel to gain insight into the perspectives of key decision-makers across the projects. Interviews allowed for additional exploration of the challenges and key lessons learned from the application of the biomimetic design tools. While published project documents are typically information-rich, interviews allow for further exploration of the ‘what’ and ‘why’, and investigation of facets of the project which may not have been highlighted in the published literature.

Interview participants included employees engaged in the application of biomimetic design and engineering on the case-study projects. All participants were 18 years of age or older, however no other personal attributes were specified. All subjects gave their informed consent for inclusion prior to participating in the study, and the research was conducted in accordance with the methodology approved by the Ethics Committee of Griffith University, Queensland, Australia.

Criteria for selecting participants involved in the case-study projects included:

- Direct involvement with the application of biomimicry on the selected project; and
- Decision-making role relevant to the biomimetic application; and
- Availability, willingness and approval for involvement.

Potential participants were identified through relevant publications and were asked to suggest additional personnel who may offer valuable insights into the learning, benefits and challenges of the project. The primary deciding factor for participant numbers was the point at which interview responses suggest that saturation had been reached, and that no new materially relevant information was being obtained as a result of additional interviews [24]. 

The interviews adopted a structured interview technique with a list of pre-defined questions to support consistency across all interviews [25]. The objective of the interviews was to validate findings from the literature and source additional information, as well as to obtain participant perspectives on drivers, learning, and outcomes. Potential participants were contacted initially via phone or email, with follow-up contact by telephone and/or video call. Interviews were conducted by videoconference and interview duration was approximately one hour. A total of 13 participants were interviewed across the case-study projects, resulting in 13 interviews in addition to 9 scoping and project discussions and 39 supplementary interactions.

All interviews were audio-recorded and transcribed to word documents, before being uploaded into NVivo qualitative data analysis software (Version 12, 2018, QSR International Pty Ltd., Melbourne, Victoria, Australia) for further analysis. Initial coding was conducted against the following nodes, informed by the research question and interview design: (1) Benefits; (2) Challenges; (3) Drivers: (4) Frameworks and Standards; (5) Learning; (6) Network focus areas; (7) Opportunities for application in infrastructure; (8) Other comments; (9) Recommendations; (10) What worked well. These categories were reviewed for key emergent themes, and subcategories were developed to inform subsequent coding.

### 2.3. Document Analysis Method

Document analysis was used as a triangulation method with interview results, and to provide greater insight into project details, process and methods. Document analysis provides an efficient method of obtaining pre-documented information, allowing for interview time to be focused instead on questions benefiting from more subjective or interpretive insights. Given that each of the case-study projects had documented their process and/or outcomes in some form, and that projects were willing to share this information, such analysis offers a logical method of data gathering and triangulation. 

Documents were requested via email, with a list of prompts included to guide document selection and provision. The document prompts were designed to elicit documents that provided adequate detail on the project scope, process, and outcomes, with a focus on lessons learnt, and benefits and challenges, as informed by the overarching research question. A total of 16 documents were reviewed across 6 case studies. These included detailed project reports covering objectives, process, outcomes and lessons learnt. Additional assessed items included ‘Request for Proposal’ submissions, project funding documentation, internal project notes, a master’s thesis and a design report.

At least one substantial and relevant document was obtained for each of the case-study projects. Within this, there was some variability in completeness. One project, for example, had a customer facing document that largely outlined results for use by practitioners, with limited coverage of limitations and challenges. Nonetheless, it was deemed that the documents analysed provided sufficient depth and coverage for coding against the key areas and themes selected. Document analysis was conducted in NVivo (Version 12, 2018, QSR International Pty Ltd., Melbourne, Victoria, Australia), with a first pass review of content for suitability and relevance, followed by detailed coding and identification of emerging themes. 

### 2.4. Analysis Across Interviews and Documents

Following coding of all interview and document transcripts, a summary of key themes was constructed and then analysed to identify patterns, similarities and differences in content and themes between the interview findings and document analysis findings. In this process, key categories were identified for further investigation. 

## 3. Results

In this section we present the case-study characteristics and the suite of 12 key themes emerging from the analysis of interview and document analyses.

### 3.1. Case-Study Characteristics

A review of applied examples of system-level biomimetic design approaches identified several projects underway or completed in built-environment applications. In total, 19 projects were identified, 6 projects selected and 13 excluded based on selection criteria outlined in Section 2.1. These are discussed in the following paragraph, referring to Table 2 which provides a summary of characteristics including their application type (planning, building, infrastructure), project code, location, project phase at which the biomimetic approach was implemented (plan, design, construct, operate, end of life), and the biomimicry tools and frameworks adopted. 

Considering the project lifecycle, the selected case studies spanned the plan, design and construct phases, however there was only one example of construction. None of the projects reviewed had reached the operational phase or explicitly addressed end-of-life considerations. Regarding use of biomimicry approaches, the projects included at least one example each Ecological Performance Standards (EPS), Life’s Principles (LP) and Genius of Place (GoP) studies. However, the selected case studies did not include examples of applying GoP or LP in building contexts, or EPS in infrastructure projects. The EPS approach was the most widely used in this project sample, within three of the six projects. However, only one of these EPS projects incorporated a second approach (LP or GoP). For projects conducting a Genius of Place study, however, all three also considered Life’s Principles.

### 3.2. Case-Study Overview

Case study 1 (CS1): this was a design project where the team identified recurring design challenges faced across their business portfolio. While it was not feasible to engage in extensive research at the individual project level, the organisation was eager to explore innovation solutions to these recurring challenges. Turning to biomimicry, the team adopted the Genius of Place design approach, to identify locally attuned design solutions drawn from local organisms and ecosystems. Given the geographically diverse nature of the portfolio projects, they opted to investigate the ecosystem type that was common to many of their project locations. First identifying design challenges, they then sought out organisms within that ecosystem type that were particularly well adapted to addressing the challenges. Drilling down to the specific design strategies of those organisms, they abstracted these to design and engineering approaches that could be considered and applied across various projects. The results were then published in a design guide for use across the portfolio and to educate a broader audience on the Genius of Place design approach. While these were later applied across various projects, the project has been classified as reaching ‘planning’ phase for the purpose of this case study, reflecting the phase of the project that is captured in the documents and interviews.

Case study 2 (CS2): as part of a broader planning and development program, the project team were engaged to advise how biomimicry could inform planning and design approaches for resilience. Life’s principles were used to inform overarching design principles, while the EPS framework was adopted to support the broader team in understanding the current ecosystem services generated by the project site, as well as the projected potential impact of business as usual development. This created a baseline for improvement, where developers could seek to reduce the overall impact of development on the provision of ecosystem services, and ideally, pursue regenerative design solutions that maximized ecosystem services—asking the question ‘what would nature do here?’.

Case Study 3 (CS3): following extreme flood events that led to damaged infrastructure, this local government authority decided to explore opportunities for ‘design inspired by nature’ in their redesign and repair. As part of the project, biomimicry experts investigated the local ecosystem to identify organisms that were particularly well adapted to shedding water, enhancing infiltration and maintaining shape under pressure. After selecting seven organisms and investigating the design features and mechanisms that supported these performance outcomes, the team conducted a series of training sessions and design charettes, to identify how these locally attuned design strategies could be translated into design and construction of the access infrastructure. While a detailed design phase was not reached, design concepts were prepared specifically for the infrastructure project, and as such this project is classified as reaching ‘design’ phase.

Case Study 4 (CS4): eager to shift towards regenerative design solutions, this organisation piloted the application of Ecological Performance Standards (EPS) to inform design and refurbishments of two operating facilities. While the first pilot was largely qualitative, the second iteration included quantitative assessment of the ecosystem services that would have been provided by an intact ecosystem on that site. Following this, business as usual design was assessed to quantify the expected ecosystem services performance, and the team then investigated a wide range of products and design opportunities to reduce this gap. Ecosystem services were prioritized in response to data availability, ecosystem characteristics and business priorities. These projects were considered as pilot projects toward a broader objective of designing facilities that contributed to, rather than detracting from, ecosystem service and function.

Case Study 5 (CS5): this project was initiated by a builder with a keen interest in biomimicry, who was interested in developing and refining the EPS framework for use in built-environment projects. It involved detailed exploration of how the EPS approach might apply to a small-scale building project, as well as identification of ecosystem services and extensive investigation of primary literature, historical datasets and other relevant sources to inform the development and quantification of ecosystem services for the project site. While the project did not progress to detailed design, it generated material contributions to the EPS framework and methodology.

Case Study 6 (CS6): this case-study project involved an ecosystem study using the Genius of Place methodology. The project team investigated the selected ecosystem to identify organisms well adapted to the design challenge of managing stormwater. Engaging local experts and practitioners, seven biological strategies were selected, design strategies extracted, and design concepts brainstormed for built-environment applications. A key output of this project was the development and publication of detailed project reporting and process guidelines, as well as detailed insights into challenges and lessons learnt, with a goal of building a foundation for future practice.

### 3.3. Thematic Coding Themes

In investigating challenges and priorities for system-level biomimicry in built-environment design, 12 key themes emerged from the thematic coding as described in the following paragraphs. These are also summarized (in alphabetical order) in Table 3, including the contribution to theme development from interview and case-study data (represented as numbers of pages coded to each theme).

**Changing worldview:** in both the interviews and document analysis findings, there was recognition of the role that broader societal and industry contexts play in creating the conditions for success in these projects, beyond project-specific content and design approaches. Interviewees referred to an understanding of the built environment as part of the surrounding ecosystem, where *“the structure of a building and a site is part of the structure and function of the surrounding ecosystem”* (CS5), and, *“the connection of built and natural environments, recognising that living systems are “complex, adaptive systems in dynamic non-equilibrium”,* capable of self organisation, renewal and adaptation (CS5). This context provides a foundation for place-based design, including learning from the history of the place, with ‘nature as mentor and model’ to achieve locally attuned and sustainable design. The conceptualization of development and growth within dynamic contexts included an understanding that such development requires an ability to adapt to changing conditions over time and that the expectations of built-environment design will continue to change in line with shifts in public perceptions and standards.

**Cost/funding:** both the interview and document analyses highlighted references to cost and funding challenges in relation to funding the case-study projects, from availability of funding sources to budget overrun. Barriers to funding included, for public sector organisations, variability in the availability of tax-payer funding. In multiple cases, projects relied on pro-bono and volunteer efforts in order to support the project execution, often relying on enthusiastic project champions—*“the team cheerfully volunteered the hours to deliver high quality products”* (CS6). Budget overruns included unexpected expenses associated with the time required to frame and implement the biomimetic design process, particularly for pilot projects, with one interviewee noting *“Because it’s a pilot project it will cost more money, it will take more time, and we are a taxpayer-based organisation so we have to be credible and fiscally responsible with taxpayers money”* (CS3). For infrastructure applications, the high cost of innovation was identified as a key challenge, due in large part to the scale and long lifespan of infrastructure assets.

**Developing metrics and benchmarks:** establishing ‘Proof of concept’ was a key driver across multiple case-study projects, with an eagerness to test, refine, and provide evidence for the frameworks and metrics adopted, and a view to standardizing metrics and creating a catalogue for future projects. Various barriers and challenges emerged when quantitative metrics were attempted—“*The vision is still very strong, but I think now we know a little bit more about the intensity of the design challenge that comes with it.*” (CS4). Technical limitations included the availability of scientific literature to support the development of ecosystem baselines or performance metrics. Where information was available, it was at times in formats aligned with qualitative rather than quantitative metrics, thus project teams were required to navigate a balance of quantitative and qualitative metrics and performance targets. Interdisciplinary translation of the data and lexicon was also a challenge. Typically, project teams were required to investigate biological and ecological literature to identify information, data and metrics, before translating this to metrics and targets that could be understood and used by built-environment professionals. Limitations in available data often meant that the selected metrics were influenced more by information availability and ease as opposed to level of materiality or relevance for the project. 

**Engagement and education:** several case-study projects demonstrated a strong focus on engagement, citing the importance of cross-disciplinary engagement and collaboration, including the establishment of a shared vision. For the most part, the focus was on internal engagement, ensuring that employees across the organisation were appropriately engaged and informed of the biomimetic design approach—*“It was a huge educational opportunity for so many people to actually just learn what the field of biomimicry is and what it’s trying to do. And I think it definitely stimulated a lot of thought for how it could be applied outside of that specific project…”* (CS3) and *“What [Company A] has really helped me see, is how this is not just an engineering challenge. It’s a employee engagement challenge as well”* (CS4). Workshops were widely used as engagement mechanisms, both to educate staff and external stakeholders on biomimicry and the biomimetic design process, as well as to conduct design charettes and collaboratively brainstorm biomimicry opportunities and design solutions for the project. Where engagement was not a project priority, this was identified by the project teams to be a limitation that could be improved in future iterations—*“If we had the time and the staff, I would have gone back and made it a bigger deal”* (CS4). It was also noted that while up-front engagement was strong in some projects, opportunities exist to plan for ongoing engagement, involvement and cultivation of interest after initial workshops. 

**Frameworks and Governance/Guidance:** in looking at enabling mechanisms and opportunities for broader uptake moving forward, projects identified the opportunity to align with building codes that support innovation (*“building codes need to evolve towards an ecological building code that does represent the limitations of an ecosystem and those built systems that are intertwined within that system”* (CS4), and to link in with regional strategies, plans and policy positions—approaches already adopted in a number of the case-study projects. Transport agencies and natural resources/forest services were identified as key agencies at present, however it was noted that there exists a significant opportunity for city-level strategies to drive engagement, set priorities and establish requirements around these biomimicry frameworks and approaches “*because then the design can really have context, when the city… adopts it*” (CS4). Current approaches often included aligning with corporate strategies, however additional opportunities exist to link in with rating schemes and frameworks such as the Living Building Challenge [26] and the Sustainable Sites [27] initiatives. 

**Knowledge-sharing** (Information availability and accessibility): limits to the availability of scientific literature and government information presented important and recurring challenges across the projects. Given the pilot nature of these initiatives, there was typically limited information available on prior application of the frameworks and tools in similar contexts. Significant resourcing was required to source, verify, filter, prioritise and translate scientific data and information into project-ready data—“*The challenge that we had on this particular project that I hope we wouldn’t have as much on future projects is a lot of the available time was spent on research and not in my opinion on design*” (CS4). Moving forward, recommendations included the development of a centralized database to support the applications of EPS, including ecosystem service information such as definitions, categories, and examples of qualitative and quantitative metrics and performance targets, to provide “*some anchor, for everybody who wants to do this to say ‘This is how we’re going to define the metrics*” (CS4). Furthermore, a best practice database organized by ecosystem service would help to consolidate tangible approaches to supporting ecosystem services through design and construction. Similar recommendations were made for the Life’s Principles and Genius of Place frameworks.

Multiple participants noted opportunities to create a more cohesive and consistent approach moving forward. This could be supported by sharing of lessons learnt, outcomes and process approaches. Project teams noted that data on prior projects, including cost/benefit analysis (“*the availability of actual numbers of cost savings*” (CS3)) and tangible performance outcomes, would enable them to build a more robust business case for adopting the biomimetic design process, as well as evidencing proof of concept and application—*“They want to see that it’s been done somewhere else”* (CS3).

**Mainstreaming:** documentation of methodologies and publication of project outcomes, including a list/taxonomy of best practices and opportunities was identified as a key opportunity to support mainstreaming of system-level biomimetic design approaches—*“I would love to have this…list of best practices and green design organized by…ecosystem service for example.”* (CS4) or *“an app that can be easily accessed by builders”* (CS5). Limited scaling has been achieved to date within the organisations involved in the case-study projects, and several challenges were identified, including the applicability and transferability of tools, data and processes across project types and locations. The inherently ‘place-based’ nature of these frameworks presented challenges to scaling and mainstreaming within organisations, with on interviewee noting *“The more you take the actual key goals and metrics and move them to another place, you immediately impact the ability to perform to that”* (CS4), however efforts were underway to address these and strike a balance between scalability and tailored solutions. Teams again noted the opportunity for collaborative knowledge-sharing to support broader uptake and mainstreaming of these approaches, as well as supporting the establishment of best practice guidance and communities of practice.

**Market supply/demand:** attempts to engage the industry in the biomimetic design process were met with varying levels of success. Participants in one project noted the difficulty associated with writing and framing the request for proposal (RFP) for a biomimetic design brief (*“it took us a year to write the RFP because there weren’t a lot of examples, and we couldn’t consult with [Company B] about it, because we wanted to give them the ability to bid”* (CS3), as well as challenges experienced by the market in responding to such RFPs. Contributing factors included the limited experience of the market in delivering biomimetic design solutions, the limited availability of biomimetic products and technical solutions, and the somewhat limited practitioner and expert networks to call on. In a given location or discipline, for example, the small number of biomimicry professionals could mean that all relevant experts are in fact tendering for the project, leaving none of these professionals available to provide mentoring or guidance to the organisation scoping and developing the RFP. Nonetheless, the process was seen as an important early step in introducing key concepts, piloting engagement approaches, establishing an organisation as a thought leader and beginning to articulate a vision for the industry.

**Organisational culture:** interview findings highlighted organisational culture as important to both the successes and challenges of the case-study projects, with 11 interviewees across 5 case studies making specific mention of this aspect. Challenges included conflict between regulators and developers, as well as internal conflict within project teams and organisations. In some cases, the risk-averse nature of engineering disciplines was highlighted as a key challenge to overcome. This included a strong cultural predisposition towards least cost, highest efficiency and lowest risk approaches—*“how do we do the minimum or most efficient minimum amount of work for a predetermined result, and I don’t think that leaves a lot of room for creativity”* (CS3). In one organisation with a well-established innovation culture, it was noted that the organisation allowed the project the space required to invest in research, exploration, scoping and process refinement without seeing this additional time investment as a project failure, as it was perceived on other case-study projects. According to one interviewee, *“What we’ve been seeking is buy-in, to fund and explore an idea, which in our company is not challenging”* (CS4), and an external consultant from that project stating that the company *“has a real strong culture of empowering the individual*” (CS4). 

**Project management:** regarding project management, internal engagement and support was noted as both a priority and a challenge, with difficulties in managing communication and working relationships between different disciplines, including ecologists and engineers, who may not often typically collaborate in detail on design projects. Once established, however, it was noted that these cross-disciplinary relationships provided significant and tangible benefits to project delivery and the identification of design solutions. The importance of targeted team-building exercises was also recognised, including early establishment of connections and interactions between team members and identification of team members’ strengths, skills and levels of expertise. Recommendations also included leveraging key individual skills, clearly defining accountabilities and engaging appropriate experts from each relevant discipline. Respondents noted the importance of engaging trained biomimicry professionals to provide biomimicry advice, training and facilitation throughout the project. Handover from design teams to engineering, construction and operation teams was cited as a priority area that, if managed poorly, resulted in a significant loss of knowledge and expertise across the project cycle. 

**Scoping and boundaries:** establishing the project boundaries and scope was repeatedly highlighted as a project challenge. In many cases, this was due to the complex, adaptive and dynamic nature of ecosystems, where it was impractical or unfeasible to delineate the project site from its upstream and downstream ecosystems and built environments, or to reflect seasonal or other temporal changes and boundaries. Reflecting systems complexity was identified as a key challenge, in particular for EPS, where it was recognised that while efforts were made to reflect ecosystem cycles and interrelationships, final boundaries and metrics were necessarily reductive.

The importance of clearly defining *“a realistic scope at the start”* (CS3) of the project was referenced as a key learning. Design workshops that engaged a wide range of stakeholders and disciplines often generated a multitude of design ideas and opportunities; however, many were deemed to be unfeasible, outside of budget or unaligned with technological or market readiness. For these reasons, early clarification of design parameters, limitations and boundaries were proposed for future projects. This extended to accepting the varying degrees of biomimetic solutions, from entry-level to ideal, recognising that the ideal solutions may not be feasible in the first iteration. One interviewee highlighted that while such gradients are accepted within green building and infrastructure (e.g., the varying levels of achievement in industry rating schemes), opportunities to pursue ‘partial’ or ‘limited’ biomimicry were unclear and could result in solutions that were not truly biomimetic. Balancing scientific and technological rigour with project and organisational limitations and resource constraints was identified as a primary challenge across the case-study projects. While participants recognised the need for streamlining, setting boundaries and making assumptions in the absence of robust data, there was debate around the extent to which this could occur before the robustness and defensibility of the project was jeopardized.

**Sustainability leadership:** the interview findings highlighted that a strong desire for sustainability leadership was a prominent motivating factor behind multiple case-study projects. This included a desire to leave a “sustainability legacy” as well as an eagerness to help shift respective industries and sectors forward by piloting and providing proof-of-concept for new and emerging ideas and approaches—*“we individually can’t solve the challenges of climate change, we need to share our thinking with other people”* (CS1). Alignment with corporate values around sustainability and environmental protection were also cited by interviewees as drivers for adopting the biomimetic design approach, as well as an eagerness to shift beyond business as usual approaches and to push the boundaries of current sustainability efforts. For example, *“They want to be thought leaders, and not only thought leaders they want action, and they want to be truly leaders in the field”* (CS4). Reputational and marketing benefits of sustainability-oriented projects were also recognised and identified as important benefits of adopting a leadership position, particularly where such projects often required additional up-front resourcing and investment.

## 4. Discussion

In this section the authors present an emergent conceptual model of the findings, drawing on existing theoretical framing provided by Geels [28] and Nan [23] in innovation pathways and diffusion. The model is then used to discuss the themes identified from the case studies, with regard to: innovation cultures and their influence on biomimetic innovation; opportunities that arise in transitions within maturing markets; the importance of knowledge transfer between innovative companies and the market; and potential next steps for stakeholders within the built-environment sector.

### 4.1. A Conceptual Model of Findings

**Figure 1:**
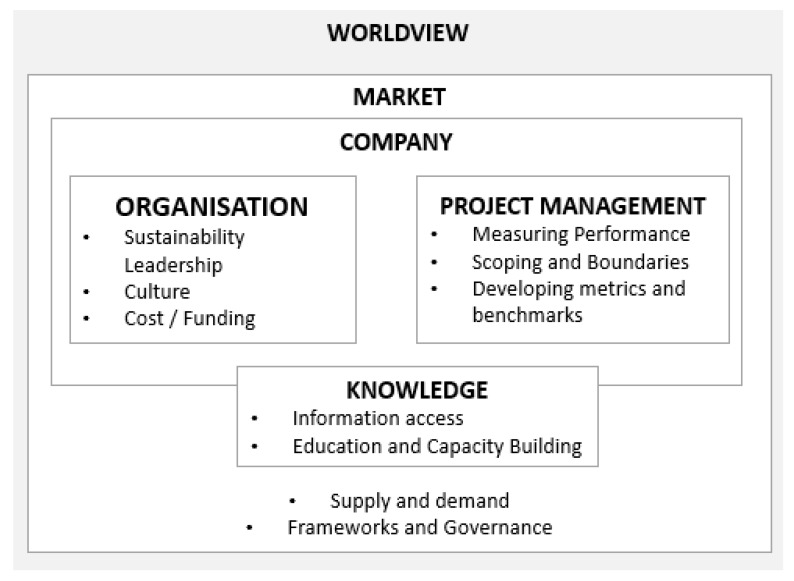
Context and relationships towards mainstreaming.

Looking to [22] the innovation framework, we see three key levels that must align in order for niche innovations to move to the mainstream. At the meta level sits the global context and trends (exogenous socio-technical landscape); at the mid-level, the status quo operating practices and ‘rules’ for industry (socio-technical regimes); and at the ‘niche’ level, technological niches of innovation, or ‘the locus for radical innovations’ [28]. Drawing from the key themes identified in Section 3, Figure 1 situates these within a model guided by these levels, reflecting themes relating to global context, market and organisation/ project levels. This includes organisational considerations of sustainability leadership motivations, culture and the availability of funding that emerged across the case studies, with important project management lessons around performance measurement, scoping and benchmarking. An emergent commonality across the projects was a recognition that documentation and sharing of learning from individual projects would enable the establishment of a community of practice and best practice guidance that could then support mainstreaming and a shift in the broader market context. 

#### 4.1.1. Innovation Cultures and Their Influence

As is familiar in many areas of sustainability, regenerative design and innovation, a recurring message across the case-study projects was the importance of passionate individual project champions (or innovation agents)– those who would continue to push, promote and pursue the initiatives in spite of barriers such as lack of broader engagement and leadership, and limited resourcing. These influencers developed, fostered and communicated the innovation niches. Although such champions are integral in pushing the boundaries for innovation, there are limits to the ability to sustain such enthusiasm over the long term, unless supported by an organisational culture that creates the space and opportunity for iterative and collaborative development of emerging ideas. 

In infrastructure applications, low-risk organisational culture and the desire for low-cost, proven options to demonstrate success, were identified as barriers, recognising the challenges of doing so when there are limited project examples. An approach focused entirely on pursuing the most cost-effective minimum to achieve the pre-determined result limits room for piloting and testing innovative approaches, with creative leaders unable to secure the support and resourcing required to explore unique approaches. One case study was conducted within a large corporate organisation with a strong and intentional innovation culture, where structured frameworks, approaches and funding mechanisms had been established to support innovation. As outlined by Nan [23], when introducing innovative approaches in complex systems, success relies not only on individual champions, but the capability stocks required for other agents to implement the innovative solutions. This capability includes knowledge and understanding, as well as the availability of funding and other resources. Further, the organisation must have robust networks that foster interaction across levels, across disciplines, and with both internal and external actors, where projects can also meaningfully link to external market priorities and broader industry shifts. With biomimicry requiring interdisciplinary collaboration (e.g., between biologists and engineers), project success may be severely limited by inflexible siloed operating practices, particularly where there are limitations to interaction and feedback across levels and disciplines.

#### 4.1.2. Transitions in Maturing Markets

The absence of proven examples to draw on when building a business case for approval was a recurring challenge. This included limited biomimetic products, technologies and services available in the market, as well as challenges in accessing information on prior projects. Multiple interviewees referenced the opportunity to create a centralised database of project information as a tool for the market, as well as motivating broader uptake by communicating project outcomes and benefits. Looking to the framework provided by Nan et al. [23], the current lack of information and market maturity reduces understanding of ‘relative advantage’ and increases the level of ‘arduousness’ associated with implementing the biomimetic innovations. One approach adopted was to categorize biomimetic opportunities into three groupings: (1) Practical application: potential for immediate implementation using existing products or approaches; (2) Conceptual but feasible: developed as a concept, but without available technology in the market (technologies exist, but not yet commercialized or scaled); or (3) Theoretical but possible: Recognizing varying levels of maturity within the field and allowing for exploration of abstract or aspirational solutions—*“a 2030 or 2050 vision”* (CS1).

Moving forward, it may be that the biomimicry field and associated networks develop unique pathways to explore each of these three opportunity types. For example, opportunities with potential for immediate implementation may be consolidated into a centralised database as suggested (leveraging, for example, the work undertaken by the Biomimicry Institute with ‘Ask Nature’ [29])—work that has now been initiated by Pedersen Zari and Hecht, through the creation of an online interactive database that highlights design interventions to support ecosystem system services [30]. Conceptual but feasible opportunities could be promoted for early piloting, and theoretical but possible opportunities earmarked for further academic and interdisciplinary investigation.

#### 4.1.3. Knowledge Transfer between Company and Market

Piloting new technologies and approaches requires an investment of time and resourcing in order to test and confirm what strategies work, what key challenges arise and where the material costs and benefits may lie. Pilot projects involve stepping out ahead of business as usual and learning the lessons that will assist in streamlining and refining methods for those that follow. As such, a common finding across the case-study projects was that these early efforts were particularly resource intensive (arduous), and that such overheads (time, cost, personnel), would need to be significantly reduced for any meaningful scaling and mainstreaming to occur. Moving forward, knowledge-sharing will be pivotal to supporting broader uptake and reducing the upfront time and resource investment required to undertake projects using these tools and frameworks. At present, each project is required to scope and establish the process and methods; conduct extensive research; identify potential solutions and research market maturity; engage partners; implement; measure success and communicate outcomes. By sharing information on project methodologies, products and design solutions, scientific and market research, metrics used, and key lessons learned, it will be possible to reduce the upfront research burden, including the groundwork required to establish a business case for action. An opportunity exists for academia, public and private actors to share the workload—for example with industry highlighting key challenges (e.g., translation of biological research into project-ready information) to be addressed by academia. Public sector participants and departments can then support uptake with mandates and project specifications, with private sector collaborating to pilot and engage with academia to support robust measurement and reporting on outcomes. 

The availability of cost/benefit calculations was recognised as a vital enabler for future projects. Noting private sector commercial sensitivities, there is an opportunity for the public sector to lead here, with transparent piloting and knowledge-sharing, while recognising significant pressure to efficiently allocate taxpayer funds. 

### 4.2. Standards and Frameworks to Create a Common Language

In the early stages of developing new approaches and frameworks, it is important that there is room for pilot projects to test, experiment and adapt, before strict standardizations or specifications are introduced. Limiting this early trial and error can lead to a ‘locking-in’ of immature approaches and methodologies before sufficient refinement and adaptation has occurred. Nonetheless, there are opportunities to ease the administrative burden of implementation through consistent and easily accessible knowledge-sharing across academia, public and private sectors—sharing methodologies, processes and outcomes in a way that allows for future projects to build upon prior work and leverage prior learning. Similarly, while it may not be ideal to mandate methods and processes, it is possible to create prompts and incentives within formal schemes, standards and codes that promote uptake and testing of these system level approaches.

As emerging biomimicry tools and frameworks shift from pilots toward mainstreaming, opportunities arise for knowledge-sharing to consolidate into communities of practice and the development of industry guidance and standardization approaches. What began as informal knowledge-sharing among peers, may shift to more formalized networks and eventually, documented guidance on best practice approaches. While it is important to allow industries to test and adapt emerging areas of practice, the documentation of frameworks and methods and the communication of project outcomes can play an integral role in supporting consistency and scaling across projects and organisations. Ideally, this can also link into academic investigation, to allow for rigorous investigation of both approaches and outcomes. Consolidation of informal networks to more structured working groups endorsed by industry leaders can assist in pushing this work forward.

In linking into schemes and standards, one opportunity is to adopt a similar approach to that taken within the Living Building Challenge (LBC) [26], when looking to address biophilic design in the scheme. The LBC team understood that this was an important focus area, yet that practice within the built environment was still emerging, as opposed to well established. As such, there was a hesitance to dictate detailed and specific methodologies or performance requirements. The approach was to specify that projects were to undertake a biophilic design workshop and report on outcomes—an approach that supports capacity-building, testing and knowledge-sharing, and allows for the scheme to capture the wide range of approaches adopted by industry when flexibility was granted. A catalogue of approaches is created, allowing for review of commonalities, differences and learning that can then be incorporated into later revisions of the scheme’s approach. A similar approach for system-level biomimicry could involve an introduction to one or more of the frameworks and tools adopted in these case study projects, without dictating specific methodologies for implementation. Ongoing trialing, adaptation and documentation of approaches then begins to establish a ‘seedbank’ of applied practices, enabling discussion and workshopping of ‘best practice’.

Another unique approach, as highlighted by one interviewee, was the potential to strategically utilize existing elements of standards and codes, such as the International Building Code, which *“has a section in it for innovative and alternative means and methods”* (CS5). This section of the code allows for alternative materials, design and methods of construction and equipment to be used, provided that the building official finds the design to be satisfactory and compliant with the intent of the code [31]. Similarly, the inclusion of ‘innovation credits’ within sustainability ratings schemes offers a pathway for piloting of unique approaches within existing schemes (See, for example [32,33,34]).

## 5. Conclusions

Representing just a snapshot of work underway in system-level biomimicry in the built environment, the six case study projects explored here highlight innovative projects being tested across public and private sectors. As may be expected, challenges related to both content and context. In the former, there were challenges associated with the development of processes and methodologies, the accessing of information and data, and the limited availability of prior project examples. These challenges may be most effectively addressed through the establishment of more formalized communities of practice and knowledge-sharing mechanisms. Such networks would benefit from involvement of industry, academic and public sector, to allow for cross-pollination of research, practice and lessons learnt. Academia have an important role to play here, in developing the theoretical foundations for biomimicry in built-environment design, and supporting rigorous boundary setting and evaluation. Private sector practitioners, including designers and engineers, will be pivotal for championing and leading industry application of these biomimetic design tools and demonstrating success. Opportunities for pilots to catalyze broader industry uptake will be largely shaped by the extent and nature of knowledge-sharing during and after piloting. Public sector representatives and project proponents have an opportunity to include biomimetic design approaches and knowledge-sharing requirements in project scoping and specifications, to prompt consideration and use of these design approaches, in varying forms, within their built-environment projects. 

With regard to context, shifts towards complex systems thinking around sustainability are happening, albeit slowly, and these shifts (including towards regenerative design), will help to create an enabling context for system-level biomimicry approaches. In the meantime, deliberate alignment of project objectives with company and industry sustainability targets and objectives, along with the utilisation of existing innovation pathways and avenues may help to reinforce the potential contribution of biomimetic approaches and leverage broader efforts and resourcing. While it may be too early to fully standardize approaches and methodologies (if indeed that is a goal at all), an opportunity exists to introduce these biomimetic tools and frameworks within industry schemes and standards, to prompt consideration and experimentation with these approaches in a way that supports continued refinement and adaptation. Ultimately, these concepts of adaptation, flexibility and uncertainty will be integral to system-level biomimicry, which must recognize the complex, adaptive nature of both the source ecosystems and the socio-eco-technological systems into which innovations are introduced.

## Figures and Tables

**Table 1:** Summary of case-study data sources.

Case Study (CS)	Location	Documents (D)	Interviews (I)	Supplementary Interactions **
CS1-GoP	USA (global)	1 (CS1-D1)	1 (CS1-I1)	3
CS2- EPS	South Africa	2 (CS2-D1- CS2-D3)	1 (CS2-I1)	3
CS3 -GoP	USA	8 (CS3-D1- CS3-D8)	4 (CS3-I1- CS3-I4)	16
CS4-A-GoP/CS4-B-GoP	AUS USA	2 (CS4-A-D1; CS4-B-D1)	5 (CS4A/B-I1- CS4A/B-I5) *	20
CS5-EPS	USA	1 (CS5-D1)	1 (CS5-I1)	4
CS6-GoP	USA	2 (CS6-D1- CS6-D2)	NIL	2
***Totals***		**16**	**13**	**48**

* Interviews spanned both case-study sites. ** Supplementary interactions limited to scoping and project discussions, and specific requests for information/engagement. Additional email and phone interactions not included. Note—Identifiers included in parentheses, where CS = Case Study; D = Document, and I = Interviews. Case-study names reflect the primary tool or framework adopted, where GoP = Genius of Place; and EPS = Ecological Performance Standards.

**Table 2:** Summary of case-study projects and their characteristics.

Application Type	Project Code	Location	Phase/s *	Genius of Place (GoP)	Life’s Principles (LP)	Ecological Performance Standards (EPS)
Planning (pre-design)	CS1	USA (global scope)	P	✓**	**✓**	-
	CS2	South Africa	P	-	**✓**	**✓**
	CS6	USA	P	**✓**	**✓**	-
Infrastructure (design innovation)	CS3	USA	P, D	**✓**	**✓**	-
Buildings (performance metrics)	CS5	USA	P	-	-	**✓**
	CS4-A CS4-B	USA	P, D, C	-	-	**✓**
		AUS	P	-	-	**✓**

* Phases annotated as follows: P = Planning; D = Design; C = Construction; O = Operation; E = End of Life. ** ✓ = Tool/Framework applied in the case study project.

**Table 3:** Summary of emergent themes from case-study analyses*.

Key Emergent Theme	Interview Data	Document Data
Changing worldview	**✓**	**✓**
Cost/funding	**✓**	**✓**
Developing metrics and benchmarks	**✓✓**	**✓✓**
Engagement and education	**✓✓✓✓**	**✓**
Frameworks and governance/guidance	**✓✓**	**✓✓**
Knowledge-sharing	**✓✓✓**	**✓**
Mainstreaming	**✓✓**	**✓**
Market supply/ demand	**✓✓**	**✓**
Organisational culture	**✓✓**	**✓**
Project Management	**✓**	**✓**
Scoping and boundaries	**✓✓✓**	**✓**
Sustainability leadership	**✓✓**	**✓**

* ✓ = 0–4.9 pages; ✓✓ = 5–9.9 pages; ✓✓✓ = 10–14.9 pages; ✓✓✓✓ = 15–19.9 pages.
